# Microfluidic in compared with Zeta potential, MACS and swim up methods, resulted in improved chromatin integrity and high quality sperms

**DOI:** 10.5935/1518-0557.20240075

**Published:** 2025

**Authors:** Nastran Vahidi, Hossein Eyni, Fatemeh Tanhaye Kalate Sabz, Nima Narimani, Zahra Zandieh, Fatemehsadat Amjadi

**Affiliations:** 1 Department of Anatomy, School of Medicine, Iran University of Medical Sciences, Tehran, Iran; 2 Stem Cell and Regenerative Medicine Research Center, Department of Anatomy, School of Medicine, Iran University of Medical Sciences, Tehran, Iran; 3 Hasheminejad Kidney Center (HKC), Iran University of Medical Sciences, Tehran, Iran; 4 Shahid Akbar Abadi Clinical Research Development Unit (SHACRDU), Iran University of Medical Sciences, Tehran, Iran

**Keywords:** microfluidic, MACS, Zeta potential, swim-up, sperm

## Abstract

**Objective:**

Sperm parameters and DNA integrity are crucial factors in ART outcomes. This study compared four sperm preparation methods (microfluidics, MACS, zeta potential, and swim-Up) for sorting spermatozoa with normal parameters and chromatin integrity.

**Methods:**

This study evaluated semen samples from 25 couples with male factor infertility. The semen samples were divided into four portions: one prepared by MACS, one by zeta potential, the other by microfluidics, and the last by swim-up. After preparation, sperm viability, motility, and morphology were assessed based on the WHO guidelines. DNA intergrity was assessed by SDF assay, and the CMA3 staining test was used to evaluate sperm chromatin packaging.

**Results:**

Compared to other preparation techniques, microfluidic preparation significantly improved sperm parameters, including motility, viability, morphology, and DNA integrity as well as chromatin packaging (*p*-value <0.05). The results also demonstrated that sperm motility, viability, and sperm DNA integrity as well as chromatin packaging, were not significantly different after preparation with MACS and Zeta potential methods. However, the MACS and Zeta methods produced improved sperm parameters and better DNA integrity than the swimup method.

**Conclusions:**

Our results indicate that microfluidics can improve sperm quality compared to other methods of sperm preparation. When the microfluidic chip is not available, considering the similar results of sperm preparation by MACS and Zeta potential methods, it is preferred to use the Zeta method for the ART cycle due to its simplicity and cost-effectiveness.

## INTRODUCTION

Sperm preparation and selection methods are essential to obtain better-quality sperm during assisted reproductive techniques (ART) cycles. To date, new and varied methods have been introduced to sort sperm with normal parameters and DNA integrity. Nevertheless, no definitive method has been established as the most effective for preparing sperm. DNA integrity plays a crucial role in sperm fertility potential. In this regard, sperm DNA fragmentation contributes to decreased fertilization rates, impaired embryo development, and consequently reduced implantation and pregnancy rates in IVF (In Vitro Fertilization)/ICSI (Intracytoplasmic Sperm Injection) cycles (Quinn *et al*., 2018). The swim-up preparation is the most commonly used method in clinics to separate sperm with high motility and morphology. However, the quality and fecundity of sperm cannot be determined by these criteria alone (Volpes *et al*., 2016). Microfluidic (Gonzalez-Castro & Carnevale, 2019), Zeta potential (Zarei-Kheirabadi *et al*., 2012), and magnetic-activated cell sorting (MACS) (Hasanen *et al*., 2020) are some of the recently developed methods for sperm separation with appropriate parameters and low DFI in ART for infertile men. Since the success rate of ART depends on selecting high-quality sperm in terms of sperm parameters and DNA integrity, we sought to find the most effective method of sperm preparation to ensure the health of sperm DNA. In this study, microfluidic, Zeta potential, magnetic-activated cell sorting (MACS), and swimup methods were evaluated in order to determine sperm parameters, DNA fragmentation, and chromatin packaging after preparation in infertile men.

## RIAL AND METHODS

### Patient selection

The present study was performed at Shahid Akbarabadi hospital IVF center, Iran University of Medical Sciences, Tehran, Iran. Local Ethics Committee approved this study (IR.IUMS.FMD.REC.1400.010). Twenty-five infertile couples with a male factor cause (concentration<15 million/ ml, total motility<40%, progressive motility<32% and normal morphology<4%) were studied.

### Semen analysis

The semen samples were collected in a sterile container 3-5 days after sexual abstinence. After liquefaction at 37°C for 20-30 min, samples were analyzed according to the standard WHO criteria (World Health Organization, 2021). For each semen sample, macroscopic evaluations, including color, volume, viscosity, pH, and microscopic assessments, including sperm concentration, motility, and morphology, were performed. The viscosity of semen was determined by gently aspirating it into a disposable pipette and allowing it to drop by gravity. A Makler chamber was used to evaluate sperm concentration and motility, including progressive and non-progressive motilities (under a ×40 objective lens). Sperm morphology was evaluated using the Diff-Quick staining kit. The viability of each semen sample was assessed using the eosin-nigrosin staining method. DFI was evaluated by the SCD test, and the Chromomycin A3 test was used for analyzing sperm chromatin packaging.

### Sperm preparation methods

After performing the mentioned assessments on semen, each semen sample was divided into four portions and then was prepared using four sperm selection techniques, including MACS, microfluidic, Zeta potential, and swim-up methods. Processed samples were used to assess sperm parameters (motility, viability, and morphology), sperm chromatin packaging and sperm DFI.

### MACS method

MACS was used to prepare a portion of the semen using the Annexin V MicroBead Kit (Miltenyi Biotec, Bergisch Gladbach, Germany). For the MACS group, sperm samples were incubated with 20 µL of annexin V-conjugated microbeads and 80 µL of binding buffer solution for 25 min during continuous agitation. The suspension was loaded onto a separation column (MiniMACS; Miltenyi Biotec) after rinsing the column with 400 µL of binding buffer. The labeled spermatozoa (annexin V-positive) were retained in the column, whereas the non-apoptotic and viable spermatozoa (annexin V-negative) passed through it. This latter fraction was recovered and further processed (Hasanen *et al*., 2020).

### Microfluidic method

Microfluidic sperm sorting was performed with a microfluidic chip (FERTILE device, Zymot, DxNow Inc., Gaithersburg, MD, USA), this chip hasan inlet sample chamber joint, in addition to an outlet collection chamber via a microfluidic channel. As described previously, the microchannel dimensions between the inlet and outlet ports hydrodynamically constrain the migration of compromised sperm while allowing motile sperm to progress to the outlet (Zhang *et al*., 2018). The microfluidic chip was loaded with a sperm-washing medium before adding semen. 50 µl of semen was processed, and the chip was then incubated for 30 min at 37°C. The processed sample was collected from the chip outlet (Quinn *et al*., 2022).

### Zeta potential method

The Zeta potential method involved transferring the semen sample to a centrifuge tube. After washing, the tube was quickly rotated three times inside the latex glove and pulled out to conceive. Then, it was placed at laboratory temperature for one minute to allow negative charge sperm to adhere to the tube wall. Sperm was deposited in the tube with a shrilled pipette were slowly removed from the bottom of the tube. 500 µl of Ham’s medium was then poured into a tube, and then it was washed and removed sperm adhering to the tube wall, placed at the bottom of the tube, and collected with a clean pipette (Kheirollahi-Kouhestani *et al*., 2009).

### Swim-up method

In this method, after liquefaction of semen, 1 ml of semen was poured into a 5 ml flow cytometry tube and mixed with sperm washing medium (sage) in 1:3 proportion. The sample was centrifuged at 1600 rpm for 10 min after discarding the supernatant. The tube was then filled with 0.5 ml of the medium and placed in an incubator at a 45° angle for 30 min. During this period, the sperms moved from the semen plasma to the medium.

Sperms obtained from all four preparation methods were analyzed using four methods, including sperm morphology, viability, DNA fragmentation, and sperm chromatin packaging.

### Evaluation of sperm parameters (motility, viability, and morphology)

The Makler chamber and contrast phase microscope were used to evaluate sperm motility before and after each preparation method. For each sample, approximately 200 sperm were evaluated. Sperm viability was assessed by the eosin-nigrosin staining technique described by David Mortimer (Mortimer, 2020) and was examined as follows. A 10µl of pure semen was put on a slide (on a hot plate at 37°C). About 10 µl of eosin-nigrosin dye was added to the sample and was gently mixed after the 30-second sample was spread on another slide. To detect abnormal sperm viability, acrosome was observed by a light microscope with a magnification of 100 and 200 sperms were counted. Then, the percentage of live sperm was calculated. Sperm morphology was examined using Diff-Quick staining (DiffQuick kit, Avicenna, Iran). This kit consists of three components: a fixative (methanol), an eosin dye that stains basic proteins, and a thiazine dye that stains sperm DNA (Tavares *et al*., 2013). After preparing the smear and drying it at room temperature, 20 µl of the diluted sample was placed on the slide. After sequentially dipping the slides in the kit solutions for 30 seconds, each slide was rinsed with water to remove excess dye. After drying at room temperature, the morphology of at least 200 sperm per slide was assessed under a light microscope.

### DFI assessment

Sperm DNA integrity was evaluated using the Halosperm test (SDFA kit; Ideh Varzan Farda, Iran). 50 µl of 1% agarose (low melting point) was incubated at 90-100°C for 15 min. Then, it was mixed with 50 µl of sperm sample (concentration: 10*10^6^ /ml at 37°C). 25 µl of the mixture was put on a slide that was already and covered with a coverslip for 15 min was preserved at 4°C in the refrigerator. Next, the coverslip was carefully separated from the slide surface. Slides were horizontally placed in 0.08 N hydrochloric acid solution at room temperature and stored in the dark for 7 minutes. After that, the slides were transferred to a lubricating solution for 15 min. After washing in distilled water and drying, the samples were dehydrated in 70%, 90%, and 100% alcohol for two minutes, respectively. After staining the slides with Wright paint solution for 10 minutes, they were rinsed with water and examined under a light microscope (magnification *100). The spermatozoa without DFI show large or medium Halos, while spermatozoa with DFI produce small or no halos.

### Evaluation of sperm protamine deficiency

CMA3 stain (Sigma-Aldrich) was used to evaluate sperm protamine deficiency, as described by Nasr et al. (Nasr-Esfahani *et al*., 2008). Semen samples in Carnavian solution (methanol and glacial acetic acid in a 3:1 ratio) were fixed at 4°C for 5 minutes, and a smear was prepared from them. After preparing and drying two smears, staining was performed with 60-100 µl of chromomycin A3 solution (with a concentration of 0.25 µg/ml in Mc-Elvin’s Buffer containing 10 mM magnesium chloride) for 20 minutes. The slides were kept in a dark, humid environment for optimal staining quality. Under a fluorescent microscope at 100% magnification, 200 sperm per slide were observed and counted. The percentage of bright yellow sperm as CMA3+ (sperm with protamine deficiency) was examined and recorded.

### Statistical analysis

The results were reported as the mean ± standard deviation for continuous variables. Data were analyzed using IBM SPSS Statistics 24.0 (SPSS Inc., USA). The one-way ANOVA test was used to compare different groups based on the normal distribution of the variables. The graphs were designed using GraphPad Prism 7.0, and a *p*-value <0.05 was considered significant.

## RESULTS

A total number of 25 infertile men were included in the study. The semen characteristics and DNA fragmentation rate of these patients are presented in Table 1.

**Table 1:** The characteristics of patients studied; Values are presented as mean±standard deviation.

Characteristics	Mean ± SD
Age (year)	37.61±3.72
BMI	3.55±25.2
Duration of infertility (year)	3.49±2.13
DFI %	39.67±6.07
Volume (ml)	2.3±1.53
Concentration (million/ml)	13.22±3.61
Motility %	27.56±6.58
Morphology %	2.44±0.62
Viability %	39.11±8.44

### Evaluation of sperm parameters

**Figure 1:**
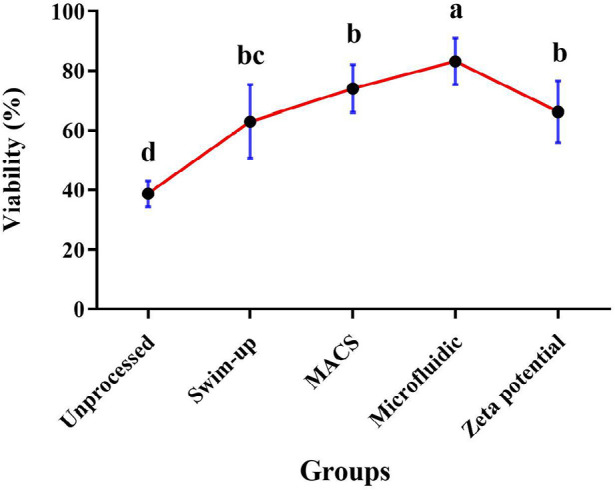
Results of viability in MACS, Zeta potential, swim-up, and microfluidic methods (*p* value<0.05); Nonsimilar letters: indicating a significant difference between sperm preparation methods; bc indicates that swim-up and Zeta potential have no significant difference.

Figure 1 demonstrates the results of the viability evaluation in four groups. The microfluidic method achieved significantly higher viability than MACS, Zeta potential, or swim-up (83.22±7.83 % *vs*. 74±8.05%, 66.33±10.38%, 63±12.39%, respectively) (Figure 1).

**Figure 2:**
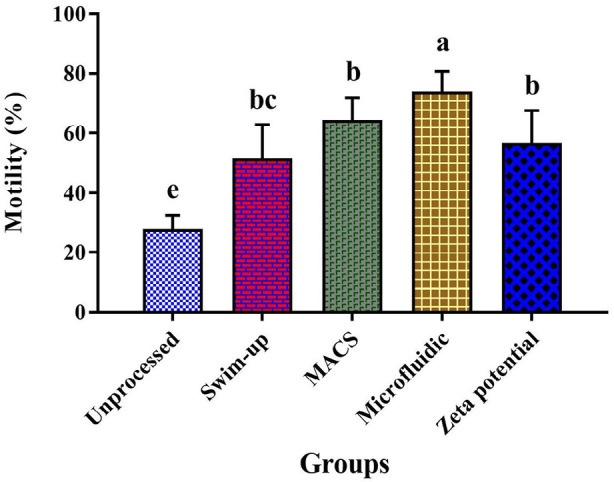
Results of motility in MACS, Zeta potential, swim-up, and microfluidic methods (*p* value<0.05); Nonsimilar letters: indicating a significant difference between sperm preparation methods; bc indicates that swim-up and Zeta potential have no significant difference.

The percentage of total sperm motility after preparation with the microfluidic method (74%±6.82) was significantly higher than semen and the other methods, including swim-up (51.39%±11.41), MACS (64.28%±7.50), Zeta potential (56.56%±10.96) (Figure 2).

**Figure 3:**
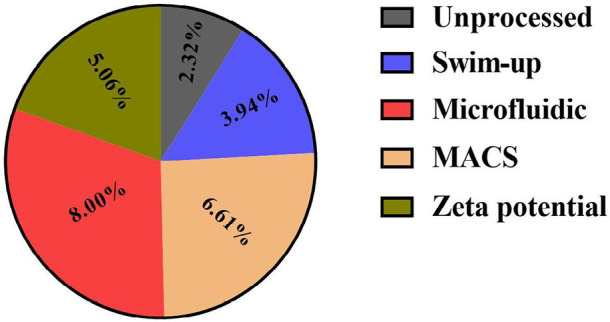
Results of Morphology in MACS, Zeta potential, swim-up, and microfluidic methods (*p* value<0.05); Non-similar letters: indicating a significant difference between sperm preparation methods.

Microfluidic preparation resulted in a significantly higher percentage of spermatozoa with normal morphology as compared to Swim-up, Zeta potential, and MACS methods (8±1.50 *vs*. 3.94±0.87, 5.06±0.94, 6.61±1.50, respectively) (Figure 3).

Compared to Swim-up, MACS showed a significant increase in sperm parameters, including viability, motility, and morphology percentage (*p*<0.05). In Zeta potential and MACS methods, sperm motility and viability had no significant difference.

### Assessment of sperm DNA fragmentation

**Figure 4:**
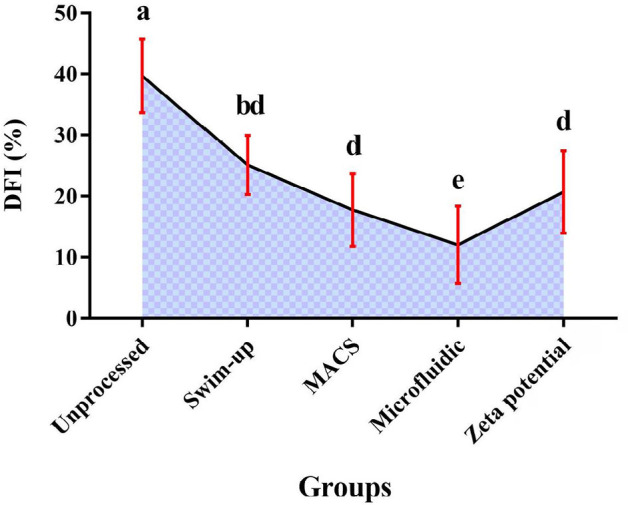
Results of DFI percentage in MACS, Zeta potential, swim-up, and microfluidic methods (*p* value<0.05); Non-similar letters: indicating a significant difference between sperm preparation methods. bd indicates that Swim-up and Zeta potential have no significant difference.

The mean ± SD of DFI data of samples were unprocessed (39.67%±6.07) and after preparation by Microfluidic (12.03%±6.36), MACS (17.72%±5.94), Zeta potential (20.67±6.76) and Swim-up (25.11%±4.84) procedures. According to these data, the microfluidic preparation method significantly improved sperm DFI (*p*<0.05) compared to other methods. Moreover, there was no significant difference in the DFI rate between the MACS and the Zeta potential procedures (Figure 4). Also, MACS and Zeta potential produced significantly higher sperm DFI than swimup (*p*<0.05).

### Evaluation of sperm chromatin packaging

**Figure 5:**
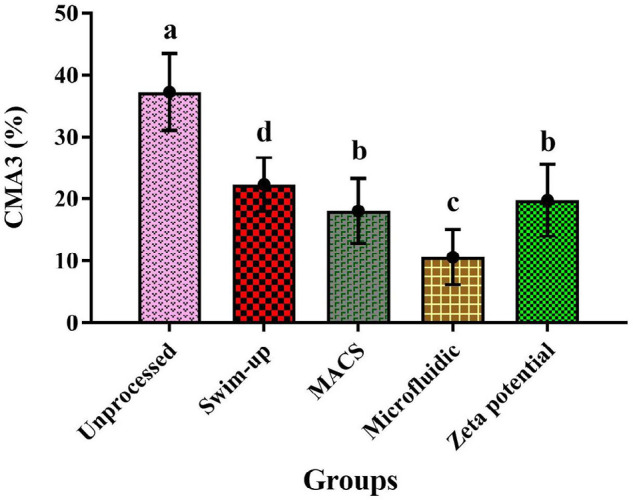
Results of CMA3 Staining for OAT men in MACS, Zeta potential, swim-up, and microfluidic methods (*p* value<0.05); Non-similar letters: indicating a significant difference between sperm preparation methods.

The results of CMA3 staining are presented in Figure 5. The mean ± SD of CMA3 stained sperm was significantly lower in the microfluidic method (10.56±4.44) than in unprocessed and other preparation methods (swim-up 22.28±4.36, Zeta potential 19.72±5.78, MACS 18±5.24) (*p*<0.05) (Figure 5).

## DISCUSSION

Nowadays, various sperm selection methods are used in the ART laboratoryto sort high-quality sperms with healthy chromatin (Li *et al*., 2016). Some studies have shown that conventional methods cause mechanical damage to sperm and increase oxygen free radicals, ultimately reducing membrane integrity and affecting the fertilizing ability of sperm (Quinn *et al*., 2018).

We evaluated some of the recently developed sperm separation methods to find the most effective one to sort best sperms. The present study revealed that sperm motility in microfluidic method was better than others, wherase sperm motility in MACS preparation method and the Zeta potential method are approximately similar. Our previous investigation showed that the microfluidic method significantly increased sperm motility compared to conventional preparation methods (Mirsanei *et al*., 2022). Several other studies have also demonstrated that the microfluidic method sorts sperms with more progressive motility (Vasilescu *et al*., 2023, Mirsanei *et al*., 2022). The high percentage of sperm motility may be attributed to the filtarion plate of the microfluidic, which prevents immobile or non-progressive sperm from passing into the outlet (Mirsanei *et al*., 2022). In contrast to a report by Zahedi *et al*. comparing MACS and Zeta potential methods, the MACS method is more efficient in selecting sperms with progressive motility (Zahedi *et al*., 2013). It was demonstrated that the total motility of spermatozoa prepared using zeta potential method was significantly higher than that obtained using swim-up (Volpes *et al*., 2016). In the Zeta potential method, the selection of natural sperms is based on the negative electric charge on the surface of their membrane. Sperms at full maturity have a negative charge on the surface of their membrane and can move rapidly and easily (Chan *et al*., 2006).

The result of microfluidic method regarding to sperm morphology showed that sperms in this group have better morpholy in comparison to others. Ozcan *et al*. highlighted the positive impact of the microfluidic method on improving sperm morphology (Ozcan *et al*., 2021). The higher rate of normal sperm morphology in this method is probably associated with the presence of filtering sheet as membrane in the microfluidic, which strictly inhibits the passage of abnormal sperm. Here, MACS significantly increased the percentage of sperms with normal morphology compared to Zeta potential and this result was matched with Zahedi et al report (Zahedi *et al*., 2013). The Zeta potential method also showed a significant improvement in the morphology of sperm in comparison to the Swim-up method, which is consistent with previous studies (Chan *et al*., 2006). Sperms with normal morphology and maturity have a normal electric potential difference, and probably for this reason, Zeta potential method separate sperms with better morphology (Chan *et al*., 2006).

Similar to motility and morphology result, sperms in the microfluidic group have highest viability wherase viability in the MACS and Zeta potential methods are approximately similar and better than swim-up group. However, previous studies have reported a significant increase in viability in the MACS preparation method compared to the Zeta potential method and it could be due to the separation nature of the MACS method that the sperms that have undergone apoptosis are separated from the viable sperms (Kheirollahi-Kouhestani *et al*., 2009).

To the extent of our knowledge, some studies have been conducted that the DFI of sperm prepared by the microfluidic method was lower than of MACS and Zeta potential methods (Vasilescu *et al*., 2023; Mirsanei *et al*., 2022). In this study, sperms preparaed by the microfluidic method had a significantly lower DFI than MACS, Zeta potential, and swim-up methods. Also, in the present study, the MACS and the Zeta potential methods were equally effective in selecting sperms with low DNA fragmentation. Nonetheless, a significantly lower DFI was obtained using the MACS and Zeta potential than the swim-up. The microfluidic method does not require centrifugation. Since centrifugation can increase ROS and result in DNA fragmentation (Kishi *et al*., 2015), removing the centrifuge step in the microfluidic method help maintain sperm DNA integrity. Centrifugation-based techniques can produce reactive oxygen species, resulting in oxidative stress and decreased DNA integrity. On the other hand, non-centrifugation-based techniques reduce oxidative stress and sort sperms with high DNA integrity (Sharma *et al*., 2015).

Similar to the current study, researchers have reported a significant reduction in the percentage of DFI in patients’ sperm prepared by the microfluidic method compared to the conventional sperm preparation methods like swimup method. Likewise, the MACS method was significantly more effective than swim up in preparing sperm with low DFI (Kishi *et al*., 2015; Hasanen *et al*., 2020). Under stressed condition, sperms undergo drastic molecular changes, which cause cell death or apoptosis. Sperm apoptosis and DNA damage are associated with symptoms such as the release of phosphatidylserine in sperm. Hence, the MACS technique is a non-invasive method for isolating non-apoptotic sperm with integrated DNA (Zarei-Kheirabadi *et al*., 2012).

Any disruption in the process of spermatogenesis leads to the plasma membrane’s underdevelopment and interferes with chromatin’s packaging. In this phase, the evolution of surface proteins in the plasma membrane may coincide with the sperm chromatin integrity. Consequently, it may be reasonable to assume that spermatozoa isolated by the Zeta potential method are likely to contain natural protamine and normal plasma membrane glycoproteins. Spermatozoa isolated by Zeta potential may have a lower percentage of DNA fragmentation because natural protamine helps package chromatin and prevent DNA fragmentation (Ozmen *et al*., 2007).

The CMA3 results showed that the amount of chromatin packaging defects in the microfluidic method was significantly lower than in other methods. The microfluidic technique does not use centrifuges, thereby minimizing DNA damage to sperm. On the other side, sperm DNA damage is directly associated with sperm chromatin packaging (Chi *et al*., 2016). It appears that the microfluidic microchannel system naturally mimics the movement of sperm in female reproductive tract. This method allows the separation of sperms with normal morphology and nuclear maturity without centrifugation (Vasilescu *et al*., 2023). There is no significant difference between the chromatin defects of sperm prepared by MACS and Zeta potential. Moreover, results indicate that the MACS method separated a higher percentage of sperms with natural protamine content than the swim-up method. The correct chromatin structure in the sperm coincides with the evolution of surface proteins in the plasma membrane. Therefore, the sperms that have more developed surface proteins, the amount of potential difference produced by these proteins is also within the normal range, and finally, these sperms have more developed chromatin (Ionov *et al*., 2020; Khakpour *et al*., 2019). For this reason, in the Zeta potential technique, which separates sperms based on the electric potential difference, the sperms that have a normal potential difference are more mature in terms of chromatin (Ionov *et al*., 2020). This explains why sperms with natural protamine content have the right glycoprotein.

## CONCLUSION

This study demonstrated that microfluidic preparation methods improved the quality of sperm and reduced DNA damage compared with MACS, Zeta potential, and the current conventional methods including swim-up. Consequently, this method is recommended to increase fertility chances, especially in male factors infertility. In the cases that microfluidic chip is not available, considering the similar results of sperm preparation by MACS and Zeta potential methods, it is preferred to use the Zeta method due to its simplicity and cost-effectiveness. Also, Considering microfluidic sorting produces lower sperm concentration yields than other preparation methods, it is not recommended for oligospermia and it’s better to be used from convetional preperation methods with improving the time centrifiguation for these pateints.

## ETHICAL DOCUMENTS AND REGISTRATION

The present study was performed at Shahid Akbarabadi hospital IVF center, Iran University of Medical Sciences, Tehran, Iran. Local Ethics Committee approved this study (IR.IUMS.FMD.REC.1400.010).
